# Fabrication of high-quality graphene oxide nanoscrolls and application in supercapacitor

**DOI:** 10.1186/s11671-015-0894-3

**Published:** 2015-04-21

**Authors:** Tianju Fan, Wenjin Zeng, Qiaoli Niu, Songzhao Tong, Kaiyu Cai, Yidong Liu, Wei Huang, Yong Min, Arthur J Epstein

**Affiliations:** Institute of Advanced Materials, Nanjing University of Posts and Telecommunications, 9 Wenyuan Road, Nanjing, Jiangsu 210046 China; State Key Laboratory of Organic Electronics and Information Displays and Fountain Global Photoelectric Technology Co., Ltd. 2 Xinyue Road, Yancheng, Jiangsu 224000 China; Department of Physics and Chemistry & Biochemistry, The Ohio State University, 100 West 18th Avenue, Columbus, OH 43210 USA

**Keywords:** 81.07.De (Nanotubes), 81.16.Be (Chemical synthesis methods), 62.23.Hj (Nanowires), 72.80.Vp (Graphene electronic transport), Graphene nanoscrolls, Shock cooling, Supercapacitors

## Abstract

We reported a simple and effective way of fabricating one-dimensional (1D) graphene oxide nanoscrolls (GONS) from graphene oxide (GO) sheets through shock cooling by liquid nitrogen. The corresponding mechanism of rolling was proposed. One possibility is the formation of ice crystals during the shock cooling process in liquid nitrogen to be the driving force. The other might be due to the uneven stress of the sheets inside or outside ice during the lyophilization. After reducing, graphene nanoscrolls (GNS) exhibited good structural stability, high specific surface area, and high specific capacitance. The capacitance properties were investigated by cyclic voltammetry, galvanostatic charge-discharge, and electrical impedance spectroscopy. A specific capacity of 156 F/g for the GNS at the current density of 1.0 A/g was obtained comparing with the specific capacity of 108 F/g for graphene sheets. Those results indicated that GNS-based rolling structure could be a kind of promising electrode material for supercapacitors and batteries.

## Background

Graphene was one-atom-thick carbon material with remarkable electronic, mechanical, and thermal properties [1,2]. It can be viewed as a fundamental two-dimensional (2D) building block for an array of nanostructures. Geim et al. described that graphene can be wrapped up into zero-dimensional (0D) buckyballs or rolled into one-dimensional nanotubes [3]. In fact, theoretical studies have long predicted that individual graphene sheets with high elasticity can undergo rapid conformational changes, which resulting in the formation of nanoscale sandwiches, buckyball, knots, and tubes via folding, crumpling, sliding, and rolling [4].

Currently, only two of the predicted structures (buckyball [5] and nanoscroll [6,7]) have been reported. Graphene nanosheets were easily crumpled into ball-like structures by capillary compression using ultrasonic atomization. In contrast to its flat structure that tended to aggregate, the crumpled graphene spheres feature had high surface area and remarkable stability against aggregation [8]. These three-dimensional (3D) bulkyball structures had demonstrated promising applications in energy conversion and storage devices [9,10]. Most of the produced carbon nanoscrolls (CNS) were rolled up by thin graphite. Recently, graphene sheet (GS)-wrapped CNS with high quality were reported by using isopropyl alcohol solution to roll up monolayer, but this method suffers from complicated procedures and low yield and throughput [11]. Therefore, the rapid development of graphene nanoscrolls (GNS) was still hindered by lacking of a simple and effective method to produce high-purity and high-quality GNS in high yield.

Another interesting one-dimensional (1D) tubular structure, CNS were formed by rolling up of flat graphite thin nanoplates [12], attracted intensive investigations as well. Due to their hybrid topology, CNS were expected to possess some unique physiochemical properties distinct from those of graphene and carbon nanotubes (CNTs). Theoretical calculations predicted that CNS were a promising candidate for hydrogen storage as they had a more accessible surface than CNTs [13,14]. In addition, the CNS possess a tunable interplanar distance by intercalation or doping. It had also been found that CNS were suitable as electrodes for supercapacitors [15] and batteries [16,17]. However, due to our limited knowledge of this new material, the research on the aforementioned graphene architectures (from graphene to nanoscroll) was limited mainly on theoretical predictions. Most of the predicted graphene architectures had not been produced experimentally.

In this work, we reported the graphene oxide nanoscrolls (GONS) of a distinct form of 1D tubular graphene architecture, which fabricated by shock cooling of aqueous graphene oxide (GO) dispersion by liquid nitrogen. The resulting stable and curly 1D structure was found to be high specific surface area and high specific capacitance. As a consequence, a high reversible capacity, good thermal stability and good rate capability, and excellent cyclic stability were achieved.

## Methods

### Chemicals and materials

Graphite powder (400 mesh) was obtained from Beijing Chemical Reagents (Beijing, China). KMnO_4_, NaNO_2_, NaOH, concentrated H_2_SO_4_, concentrated HCl, and H_2_O_2_ (30%) were all analytical grade and purchased from Shanghai Sinopharm Chemical Reagent Co., Ltd (Shanghai, China). All aqueous solutions were prepared with ultrapure water (18 MΩ).

### Preparation of graphene oxide

GO is prepared by the modified hummers method [18]. Briefly, 1.0 g of graphite and 60 mL H_2_SO_4_ (98%) was stirred in an ice bath, and 5.8 g KMnO_4_ was slowly added with stirring for 0.5 h. The solution is heated to 30°C for 2 h, 40 mL of deionizer water is added slowly, the reaction is heated to 90°C for 30 min, then, 80 mL of deionizer water is added. When the temperature is cooled to 60°C, 10 mL H_2_O_2_ (30%) is added to give an orange yellow solution. Two hundred milliliters of 5% HCl solution is added, decanted the supernatant, and centrifuged with deionizer water to pH 4 to 6, and the mixture solution is further dialyzed in a dialysis bag in 2 days, and low density large graphene oxide is obtained.

### Preparation of GNS

GO was produced by the modified Hummers method through acid oxidation of flake graphite. The primary graphite was suspended in water under sonication for 1 h, which followed by centrifugation at 4,000 rpm for 30 min and dispersed in the water. The resulting yellow mixture was cooled to room temperature. Graphene oxide was dispersed in water to form graphene oxide dispersion (0.84 mg/mL, 80 mL) in a 100-mL beaker which was rapidly cooled by surrounding with liquid nitrogen. The water was removed by freeze-drying machine. The remaining GONS (0.24 g) was dispersed in water (150 mL) and reduced by sodium nitrite (0.34 g) in a 250-mL round-bottom flask, which was then heated to 90°C for 2 h, the water was removed, and the residue was washed by water to obtain GNS.

### Characterization

Scanning electron microscope (SEM) images were obtained by a high-resolution scanning electron microscope (JEOL, JSM-7401, JEOL Ltd., Akishima-shi, Japan) at 3.0 kV. Transmission electron microscopy (TEM) experiments were performed on a high-resolution transmission electron microscope (JEOL, TEM, exited at 100 kV, JEOL Ltd., Akishima-shi, Japan) equipped with selected area electron diffraction (SAED). X-ray diffraction (XRD) was recorded on a Rigaku D/Max-RB diffractometer (Rigaku Corporation, Tokyo, Japan) with CuKα radiation at 40 kV and 120 mA. Thermogravimetric analysis (TGA) was carried out using a thermogravimetric analyzer (METTLER TOLEDO, TGA/DSC-1, Mettler-Toledo GmbH, Switzerland) from 30°C to 600°C at a heating rate of 10°C min^−1^ in N_2_. Raman spectra were characterized by a JY Horiba Raman (Aramis, Horiba, Ltd., Minami-ku Kyoto, Japan) under 514-nm laser for the accumulation intensity of three-time scan.

### Capacitance performance test

Capacitance performances of GNS materials were tested by cyclic voltammetry (CV), galvanostatic charge/discharge, and electrochemical impedance spectroscopy (on a CHI 660E, CH Instruments, Inc., Bee Cave, TX, USA). All of the electrochemical experiments were carried out in a three-electrode system. The fabrication of working electrodes was carried out as follows. GNS (4.2 mg) were mixed with carbon black and poly (tetrafluoroethylene) at a mass ratio of 85:10:5 in several drops of ethanol. The working electrode was formed after drying. The potential range of CV examination was −0.2 to 1.0 V, and that of galvanostatic charge/discharge tests was 0 to 1.0 V. Electrochemical impedance spectroscopy (EIS) tests were performed in the frequency range of 10^5^ to 0.05 Hz at 10-mV amplitude referring to open-circuit potential (OCP). Cycling stability of the materials was performed on galvanostatic charge/discharge curves at current density of 1 A/g after 2,000 cycles.

## Results and discussion

### Mechanism for the formation of GONS

**Figure 1:**
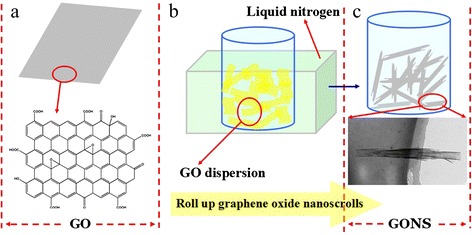
Schematic illustration of the process for the rolling up formation of GONS. **(a)** GO dispersed in water and **(b)** rolling up graphene oxide nanoscrolls with liquid nitrogen, and **(c)** the rolled up GONS.

The transformation from 2D graphene sheet into the curly tubular 1D structure could be understood with the aid of the illustrations in Figure 1. GO sheets was prepared by the chemical exfoliation from graphite. Initially, GO maintained flat sheets containing functional groups of carboxyl, hydroxy, and epoxy (Figure 1a). The hydrophilic groups on GO surface result its good dispersibility in aqueous solution. GO was dispersed in water to obtain uniform solution. Figure 1b schematically illustrated GO of the flat sheets, which rolling formation-like tubular structure in the manner of shock cooling by liquid nitrogen, which promoting the transformation from the original 2D flat structure gradually into 1D nanoscrolls. Figure 1c illustrated GONS were rolled up.

In this study, we attributed one possibility of the rolling of 2D graphene oxide sheets to the rapid formation of ice crystals, which caused by shock cooling with liquid nitrogen. As water starts to form ice crystals in this experimental procedure, GO sheets in the suspension were rejected from the moving solidification front [19] and forced to roll over by the ice crystals along the direction of the crystallization. This frozen casting method has been widely used for the production of porous structures that were made of nanoparticles [20,21]. In a latest study, Li et al. had adopted the technique to form a cellular monolith with high concentration of partially reduced GO and some fibrous structures with low concentration [22]. In our experiment, when the suspension contained flexible GO nanosheets, the frozen casting in liquid nitrogen can result in the formation of a rolling 1D nanostructure around a reel due to the anisotropic crystallization of ice. In this case, the graphene oxide sheets in this 1D rolling configuration were dominated by the formation of ice crystals. More specifically, the presence of water and the rate of forming ice played a key role in the formation of the graphene oxide nanoscrolls.

While Xu et al. suggested that the rolling was formed during the sublimation of ice [23]. During the lyophilization, GO sheets will take uneven stress when part of them exposed in the air while the other was still remaining inside ice.

### Structure of the GONS

**Figure 2:**
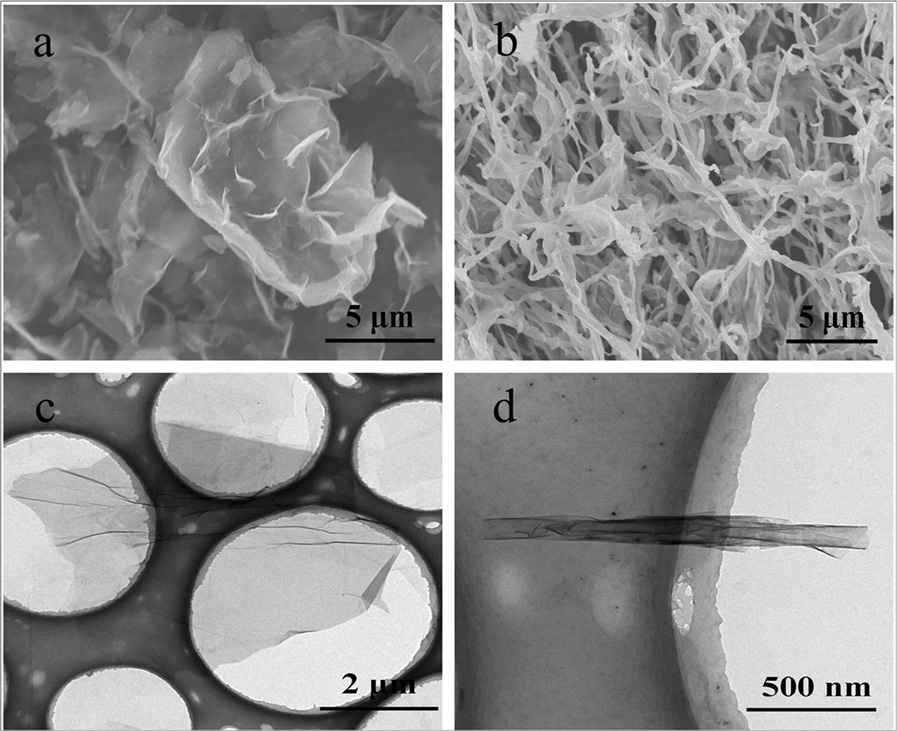
SEM images of GO. **(a)** and graphene oxide nanoscrolls with 5 μm, **(b)** TEM images of GO **(c)** and GONS **(d)**.

**Figure 3:**
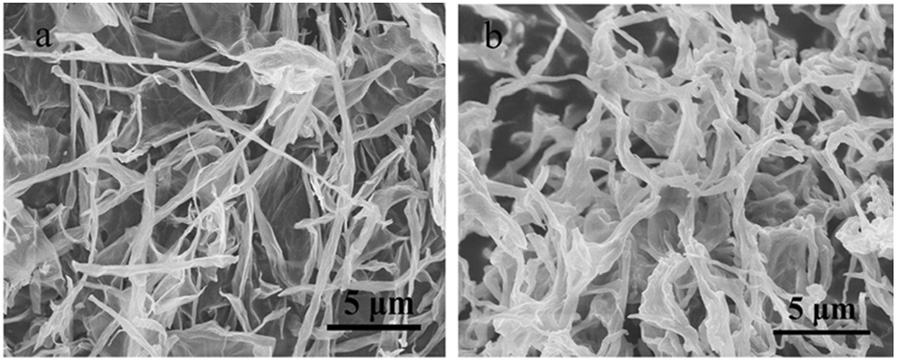
SEM images of GONS **(a)** and SEM images of GNS **(b)**.

Figure 2a,b showed a SEM image of GO sheets exfoliated from graphite. Graphene oxide sheets were crumpled into 1D tubular structure that was intertwined together. Image analysis showed that the diameter distribution of the GONS followed a normal distribution with a mean diameter of 100 nm. In comparison, freezing of GO suspension water solution only resulted in the aggregation of graphene oxide sheets due to relatively slow cooling rate and the strong π-π stacking among the GO sheets. The GONS have lengths ranging from several nanometers to several tenths of micrometers in Figure 3a. It revealed that 2D graphene oxide sheets were crumpled into a thin and curled 1D rolling structure, which likes a tubular via its closed position. However, the GONS become compressed after reducing by NH_2_NH_2_ water solution as shown in Figure 3b. This finding differed appreciably from previous experimental results where the ultrathin layers of graphene oxide tend to resemble the structure of multiwall carbon nanotubes [5].

**Figure 4:**
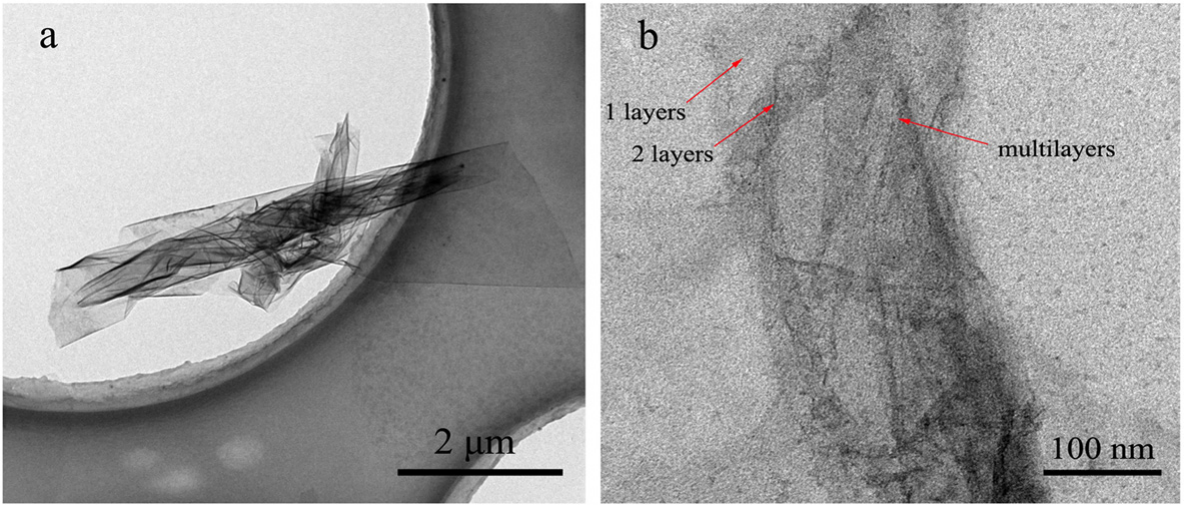
TEM images of GNS **(a)** and TEM images of a layer state in which the graphene was partially rolled **(b)**.

Figure 2c showed a typical TEM image of GO exfoliated from graphite. The GO had plat and low fold morphology. Figure 2d showed a typical TEM image of the GONS. This observation was not consistent with previous experimental studies on carbon nanoscrolls that the scrolled graphene structures tend to aggregate together [6]. This was not the close package with layers of nanoscrolls. However, the nanostructure of the individual GONS revealed a typical rolling morphology, which was formed by layer-by-layer rolling of flat graphene oxide sheets. The tubular-like rolling configurations of the GNS were further confirmed by TEM analysis (Figure 4b). A TEM image of GNS was shown in the Figure 4a, its morphology kept consistent after reducing. The stack of layers of GNS showed several interfaces, confirming the monolayer and multilayer structures. Meanwhile, it could be speculated that another striking aspect GNS were overall aligned along the direction of the rolling, potentially forming the curly axes in the rolling structure.

### Structure of GONS

**Figure 5:**
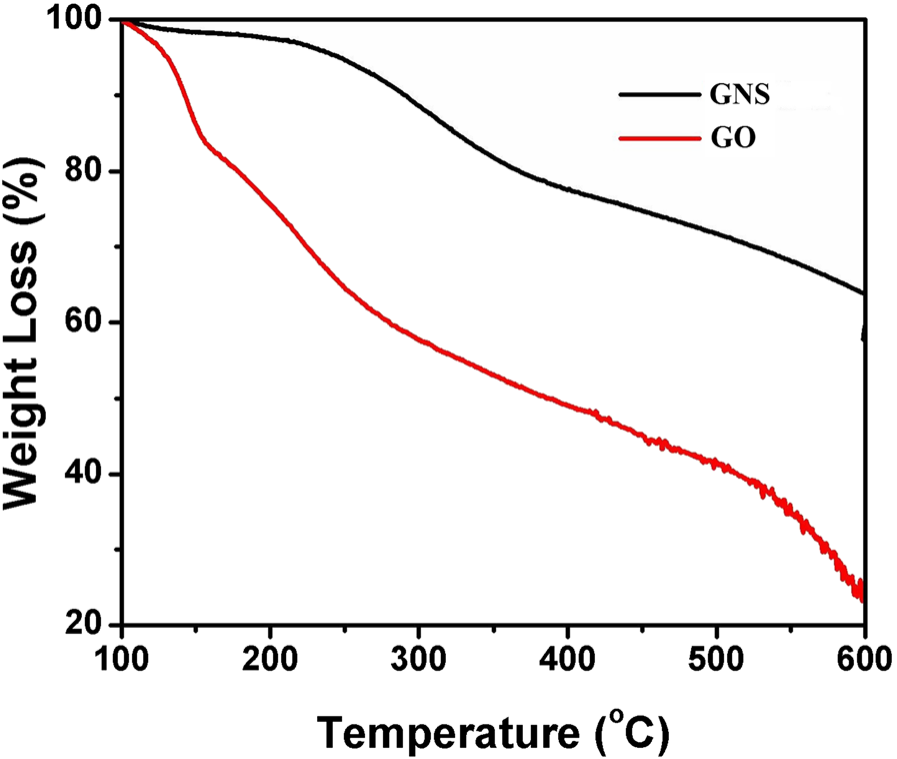
The Raman spectra of GO and GONS.

Raman spectra of GO and GONS were recorded using the 514-nm excitation line (Figure 5). The D band was related to a double resonance process involving one phonon and one defect state of the sp^2^ carbon structure. Its intensity was known to increase as the number of defects increases [24]. There was high D peak in the GO, which was oxidation exfoliated from natural graphite, which indicated it was essentially defect. GONS also produce similarly defect. However, the weak D peak observed in the Raman spectrum of the GONS might be attributed to the few defects in GONS than in GO. The Raman spectrum of the G band of GO (1,608 cm^−1^) and D band (1,365 cm^−1^) was observed with a large intensity ratio *I*_D_/*I*_G_ at 1.09. And G band of GONS (1,615 cm^−1^) and D band (1,364 cm^−1^) were observed with an intensity ratio *I*_D_/*I*_G_ at 0.917. Raman spectroscopy confirmed the quality of GO and GONS, the relative intensity of the disorder D band, and the crystalline G band (*I*_D_/*I*_G_) for as-produced GO and GONS were only around 0.179. This indicated not only the high quality of as-prepared GONS but also the differences were possibly related to the increased π-π stacking and the scrolled structure in GO.

**Figure 6:**
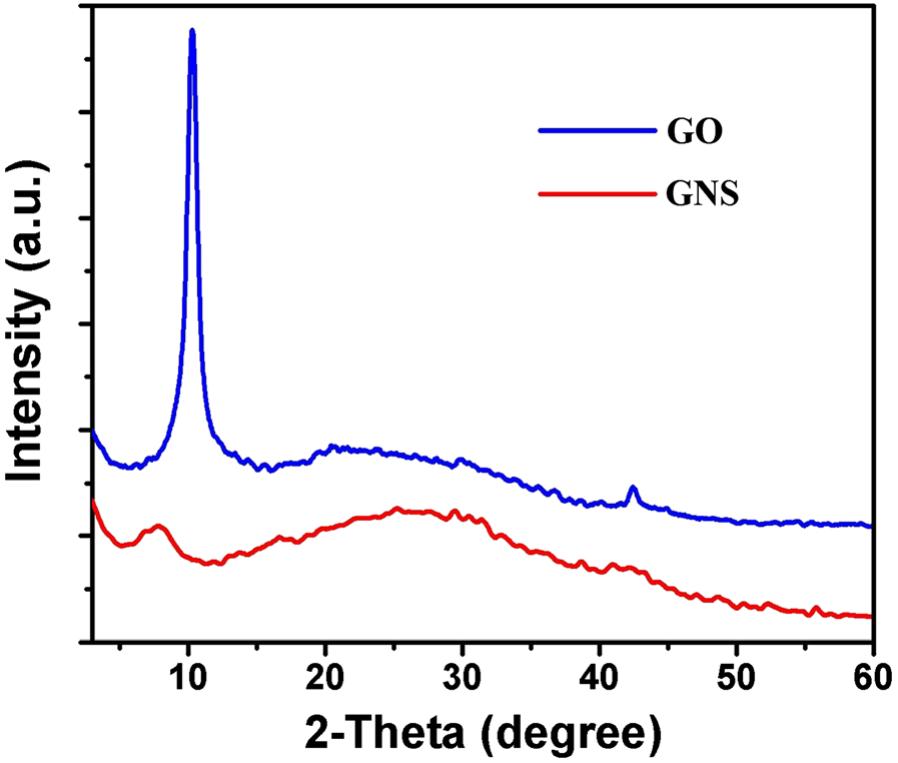
TGA curves of GO and GONS.

The GO and GONS were further studied by TGA. As shown in Figure 6, all of those materials showed a little mass loss around 100°C due to the deintercalation of H_2_O. GO showed a low mass loss from around 200°C to 400°C due to the decomposition of oxygen-containing groups [25]. The obtained GO and GONS only showed 50% and 24% mass loss from 100°C to 400°C, indicating that most of the oxygen-containing groups were removed during the chemical reduction process. TGA features suggested that the GONS showed better thermal stability with less mass loss compared with the GO. We also validated that the burning temperature of multilayer rolling graphene oxide surface was much higher. This suggested pristine GO was low-layer graphene, which was overrolling to become multilayer graphene in the forming graphene oxide nanoscroll process.

**Figure 7:**
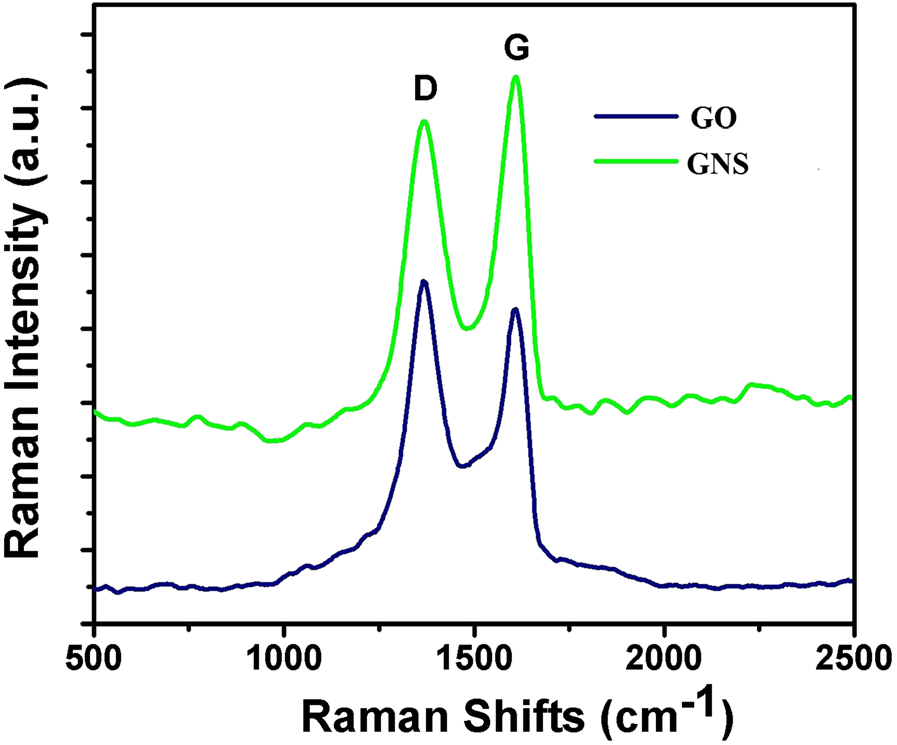
XRD spectra of GO and GONS.

In order to further evaluate the quality of the GONS, we employed XRD measurements. As shown in Figure 7, the XRD pattern of GO exhibited one peak at 10°, and GONS exhibited one peak at 8° and one broad peaks, centered at 21 to 28 [23,26], which indicated that the majority diameter of rolling graphene oxide is 5.5 Å, and the packing of GONS is much closer than the distance of GO layers.

We described the electric conductivity, density, and BET specific surface area of GO as shown in Table 1, GONS and GNS. The data showed that the rolling graphene oxide had higher conductivity, as well as higher surface area than GO. And GONS had lower density. The results were attributed to rolling graphene oxide nanoscrolls, making them intertwined together to form 3D nanonets. Thereby, the conductivity, density, and specific surface area of GONS and GNS were much higher than those of GO.

**Table 1:** **Various characteristics of GO, GONS, and GNS**

**Samples**	**Electric conductive (S/m)**	**Density (g/cm** ^**3**^ **)**	**BET surface (m** ^**2**^ **/g)**
GO	0.04	1.17	154
GONS	0.12	0.84	384
GNS	134	0.92	379

### Characteristic of capacitance properties

In order to provide more accurate measurement for practical application, the electrochemical performance of the GNS as electrode materials for supercapacitors was examined in a three-electrode system using an aqueous KOH solution (6 M) as the electrolyte [27].

**Figure 8:**
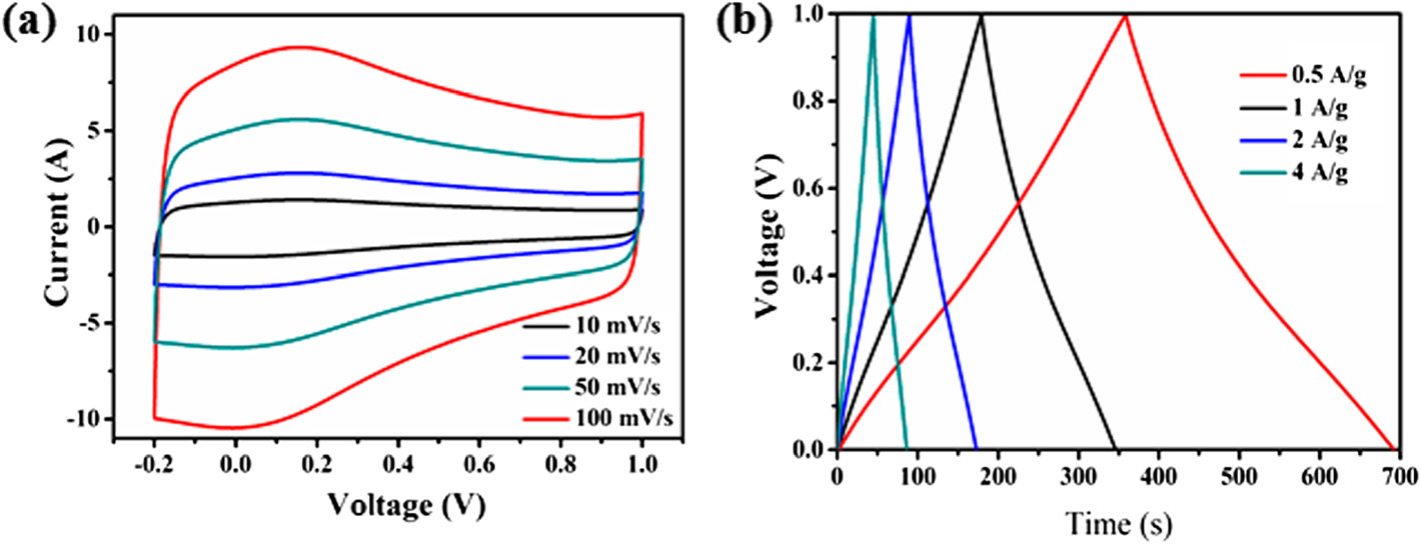
The CV curves of GNS **(a)** and charge/discharges of GNS **(b)**.

**Figure 9:**
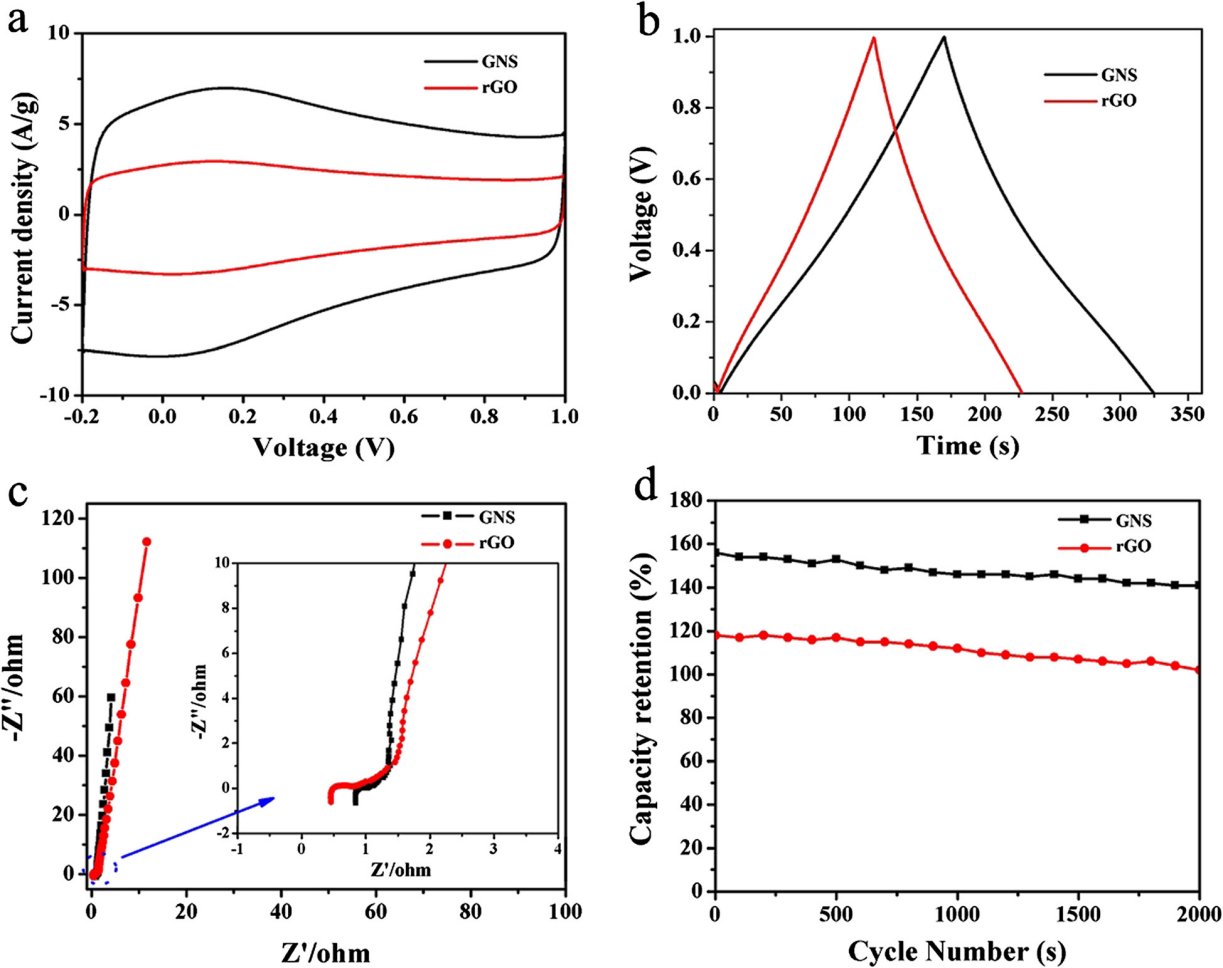
The capacitance behavior evaluation of rGO and GNS. The CV curves of rGO and GNS **(a)** and charge/discharge value of rGO and GNS **(b)**. Nyquist plots of rGO and GNS **(c)** inset showing the corresponding magnified high-frequency regions; cycle stability of rGO and GNS **(d)** during the long-term charge/discharge process at current density of 1 A/g after 2,000 cycles.

Figure 8a gives the representative CV curves of GNS with sweep rates ranging from 10 to 100 mV s^−1^. Figure 9a gives the representative CV curves of the reduced graphene oxide (rGO) and GNS with sweep rates ranging from 50 mV s^−1^. It was noted that the integrated area in the CV of GNS was larger than that of the rGO materials at the same scan rate, suggesting its higher specific capacitance. Additionally, we found that the area surrounded by CV curves form GNS electrode was apparently bigger than that of the rGO electrode at the same scan rate, implying the higher specific capacitance of GNS electrode, which indicated a remarkable contribution of rolling graphene oxide in raising the capacitance of graphene. The GNS presented well-symmetric and rectangular CV curves with obvious redox characteristic peaks indicated a low resistance of GNS electrodes to mass transfer and good charge propagation of ions at the interfaces between the electrolyte and the GNS material [28]. We found that GNS demonstrated more rectangular CV area than rGO, which further supporting the suggestion of highly capacitive nature and rapid charge-discharge behavior [29]. For rGO supercapacitors, the appearance of humps in the CV profile indicated the combination of both double-layer capacitive from graphene and pseudocapacitive behavior from the oxidation/reduction reactions of the oxygen-containing functional groups. However, there had an obvious distortion from rectangular shape when the scan rate increased to 50 mV s^−1^ in the rGO electrode [30].

Figure 8b demonstrates the charge/discharge of GNS, the curve of the GNS at different current densities of 0.5 to 4.0 A/g maintained almost the same shape in the potential range from 0 to 1.0 V, which indicated its sustainable behavior in a broad current range. Galvanostatic charge-discharge properties of plate-like samples were performed at constant current density of 0.5 A/g. The specific capacitance of SG paper decreased from 178 to 156 F/g as the current density from 0.5 to 4 A/g.

Figure 9b showed the galvanostatic charge-discharge curves of the rGO and GNS electrode collected at 1 A/g current densities. The symmetry of the charge and discharge characteristics and the almost constant slope of these curves further supported that the GNS and rGO electrode material-based capacitors had high electrochemical reversibility and excellent capacitive characteristics. The specific capacitance of the GNS was calculated to be 156 F/g at the current density of 1 A/g, which was larger than that of rGO with the specific capacitance of 108 F/g. It maybe attributable to rolling GNS which had high conductivity and high specific surface area and was comparable to those values reported for reduction of graphite oxide films [31] and heteroatom-enriched electrospun carbon nanofiber-based supercapacitors [32].

Nyquist impedance plots of the GNS and rGO electrodes are shown in Figure 9c. At high frequencies, from the Nyquist plot, the intercept at the real part (*Z*′) was a combined resistance of the ionic resistance of the electrolyte, the intrinsic resistance of the substrate, and the contact resistance at the active material/current collector interface. The semicircle in the high-medium frequency range corresponds to the Faradaic charge transfer resistance, and in the low-frequency range, it corresponds to the capacitor’s diffusive resistance of the electrolyte in the electrode pores and the ion diffusion in the host material [33]. The Nyquist plot was almost a vertical line, indicating a nearly ideal capacitive behavior of the EDLC. The inset in the Figure 9c shows the expanded high-frequency region of impedance. The electrode series resistance was derived from the high-frequency intersection of the Nyquist plot in the real axis. It was found that the GNS had higher series resistance than the rGO, manifesting the good conductivity of the electrolyte and very low internal resistance of the GNS electrode. The GNS electrode exhibited almost vertical line at the low-frequency region, indicating the desired capacitive behavior. In addition, the impedance spectrum for GNS electrode was much smaller than that of the rGO electrode, indicating the fast charge transfer process of the GNS electrode. The frequency-dependent response of supercapacitors could be analyzed from the EIS spectra. The faster performance of the GNS supercapacitor correlates with its better capacitance retention at high sweep rates in the CV measurements or higher current densities in galvanostatic charge-discharge tests. Moreover, the continuous rolling nanostructure of GNS provided a high surface area and allowed abundant adsorption of ions as well as efficient ion migration and charge transportation [34]. Thus, an exceptional specific capacitance could be realized, which was much higher than that of the more accessible reduced graphene materials. Overall, the GNS possess a significantly improved capacitance compared with the rGO and also exhibited good rate and stability performance.

Importantly, the GNS exhibited good rate capability. A specific capacitance of 156 F/g can be achieved at 1 A/g, and the specific capacitance could remain at high level of 148 F/g with a good retention above 93% (Figure 9d). On the contrary, rGO had good performance at large cycle stability. For example, when the current density was increased to 1 A/g, the specific capacitance was 108 F/g. The supercapacitor device still remained at 91.6% of the initial capacitance after 2,000 continuous charge-discharge cycles, demonstrating its good long-term cycling stability.

## Conclusions

In our work, one-dimensional GONS were fabricated by rolling GO with assistance in liquid nitrogen by a simple and effective way. The mechanism of formation was proposed, and the formation of ice crystals during the shock cooling step in liquid nitrogen were believed to be the driving force to form such unique tubular structures. GNS exhibited good structural stability, high specific surface area, and high specific capacitance after reducing GONS. Comparing with the specific capacity of 108 F/g for graphene sheets, a remarkable capacity of 156 F/g is obtained at the current density of 1.0 A/g, which was graphene sheets transformed to the unique rolling structure of GNS. Those encouraging results indicated that GONS based on the rolling structure of graphene sheets were a kind of promising material energy storage field.

## Abbreviations

0D: zero-dimensional
1D: one-dimensional
2D: two-dimensional
3D: three-dimensional
CNS: carbon nanoscrolls
CNTs: carbon nanotubes
CV: cyclic voltammetry
GNS: graphene nanoscrolls
GO: graphene oxide
GONS: graphene oxide nanoscrolls
GOs: graphene oxide sheets
GSs: graphene sheets
OCP: open-circuit potential
rGO: reduced graphene oxide
SAED: selected area electron diffraction
SEM: scanning electron microscope
TEM: transmission electron microscopy
TGA: thermogravimetric analysis
XRD: X-ray diffraction
